# Association between handgrip strength asymmetry and cognitive function across ethnicity in rural China: a cross-sectional study

**DOI:** 10.3389/fnagi.2023.1191197

**Published:** 2023-05-19

**Authors:** Wenjing Feng, Mingfeng Ma, Hanshu Gao, Wei Yuan, Ruixue Li, Hui Guo, Cuiying Gu, Zhaoqing Sun, Yao Zhang, Liqiang Zheng

**Affiliations:** ^1^Ministry of Education-Shanghai Key Laboratory of Children’s Environmental Health, School of Public Health, Shanghai Jiao Tong University School of Medicine, Shanghai, China; ^2^Department of Biostatistics and Epidemiology, School of Public Health, China Medical University, Shenyang, China; ^3^Department of Cardiology, Fenyang Hospital of Shanxi Province, Fenyang, Shanxi, China; ^4^Department of Cardiology, Shengjing Hospital of China Medical University, Shenyang, China; ^5^Department of Ultrasound, Shengjing Hospital of China Medical University, Shenyang, China

**Keywords:** handgrip strength asymmetry, handgrip strength, cognitive function, cross-sectional study, ethnicity

## Abstract

**Background:**

Recently, the association between handgrip strength (HGS) asymmetry and cognition has been revealed, but evidences are still scarce. Particularly, the association between asymmetric HGS and cognitive performance in various cognitive domains is unclear and whether this association is stable across ethnic groups is unknown.

**Method:**

The population was from a longitudinal study in rural areas of Fuxin, Liaoning, China. The Chinese version of Montreal Cognitive Assessment-Basic (MOCA-BC) was used to evaluate the cognitive function. The HGS ratio was calculated as maximal non-dominant HGS divided by maximal dominant HGS. HGS ratio <0.9 or >1.1 was classified as asymmetric dominant/non-dominant HGS, respectively. Generalized linear models were used to analyze the relationship between asymmetric HGS and cognitive function adjusted for HGS, handedness, wave, age, sex, education, ethnicity, smoking, drinking, physical labor level, BMI, hypertension, diabetes and dyslipidemia.

**Result:**

A total of 2,969 participants ≥50 years were included in this study. Adjusted for HGS and other confunding variables, there was an inverted U-shaped association between HGS ratio and MoCA-BC scores (*P*_*non*–*linear*_ = 0.004). The association between HGS ratio and MoCA-BC scores was inconsistent among ethnic groups (*P*_*interaction*_ = 0.048). In Han, only asymmetric non-dominant HGS was associated with lower cognitive scores [β = −0.67, 95% confidence interval (CI): −1.26 ∼−0.08, *P* = 0.027]; in Mongolians, asymmetric dominant HGS(β = −0.60, 95% CI: −1.35 ∼ 0.15, *P* = 0.115) and asymmetric non-dominant HGS (β = −0.56, 95% CI: −1.42 ∼ 0.31, *P* = 0.206) were all associated with lower cognitive scores, although no statistical significance was found. Asymmetric non-dominant HGS and lower HGS, but not asymmetric dominant HGS were all independently associated with impairment of Delayed Recall (OR = 1.35, 95% CI: 1.05 ∼ 1.74; OR _*per 5 kg decrease*_ = 1.10, 95% CI: 1.01 ∼ 1.21) and Fluency (OR = 1.43, 95% CI: 1.15 ∼ 1.78; OR _*per 5 kg decrease*_ = 1.10, 95% CI: 1.02 ∼ 1.19). Both asymmetric dominant HGS (OR = 1.34, 95% CI: 1.07 ∼ 1.67) and lower HGS (OR _*per 5 kg decrease*_ = 1.21, 95% CI: 1.10 ∼ 1.32) were associated with impairment of visuoperception.

**Conclusion:**

HGS and HGS asymmetry were all independently related to lower global cognitive performance. The association between HGS asymmetry and cognitive function varies among ethnic groups.

## 1. Introduction

Age-related neurodegenerative disorders, including Parkinson’s diseases (PD), Alzheimer’s disease and other dementias, are still unsolved problems in clinical therapeutics. Their increasing burden on families and healthcare systems pose a great challenge to public health (Henderson et al., 2019; Feigin et al., 2021). A latest study showed that the excess health-care cost arose as early as 10 years before a formal diagnosis of dementia (Persson et al., 2022). Therefore, there is an urgent need to understand the common pathogenesis of these neurodegenerative disorders and to identify them earlier before they occur. More and more emerging evidence has found that lower handgrip strength (HGS) was associated with not only neurodegenerative disorders (Cui et al., 2021) but also each domain of cognitive function (Sternäng et al., 2016; Firth et al., 2018; Yang et al., 2022) (e.g., visuospatial function, executive function, naming and delayed recall). A systematic review (Fritz et al., 2017) summarized the relationship between handgrip strength (HGS) and cognitive decline over time and supported the predictive function of HGS on cognitive impairment. However, these studies used different measurements of HGS. Some studies ignored the difference in grip strength between the hands (e.g., use dominant hand, non-dominant hand, maximal grip strength or other unilateral grip strength), others studies just mixed them (e.g., use the average of both hands). Few studies focused on the relationship between HGS asymmetry, characterized by wide differences in HGS between hands, and cognition (McGrath et al., 2020).

Recently, (McGrath et al., 2020) revealed that both asymmetric HGS and weakness had greater odds for lower cognitive function in American people. Chen Z. et al. (2022) found that HGS asymmetry was associated with the future risk of neurodegenerative disorders among older Chinese adults. These studies suggested that asymmetric HGS presented a characteristic that was different from maximal HGS and independently associated with cognition. HGS asymmetry may reflected hemispheric dominance in brain. A study (Sun et al., 2022) indicated that large differences in grip strength may be accompanied by significant changes in cortical representation. Furthermore, the interaction between internal sources of information, such as efferent copy and proprioception (including the grip strength) appears to be hand/hemisphere system dependent (Martin et al., 2011). The parietal and frontal areas contribute to the kinematic and dynamic of precision grasping accompanied by hemispherical lateralization, and these neural networks have been shown to contribute to language and number processing (Olivier et al., 2007). However, few population-based studies directly explored the association between asymmetric HGS and cognitive performance in specific cognitive domains and compared it to maximal HGS. In addition, people in different regions or ethnicities have different demographic characteristics and lifestyles, so that the association between HGS asymmetry and cognition may not be consistent across different populations.

The single assessment of maximum HGS can’t comprehensively evaluate the muscle function (McGrath, 2021). Promoting the use of HGS asymmetry, whose characteristics are different from maximal HGS, may advance capacity of the muscle strength assessment, and this simply requires measurement of both hands. Additional evidence of the association between HGS asymmetry and adverse outcomes will advance the implementation of the above suggestion. Therefore, we conducted a population-based cross-sectional study in Fuxin Autonomous County, a rural area on the border of China’s Liaoning province and Inner Mongolia Autonomous Region, to tentatively find whether HGS asymmetry is related to cognitive performance and specific cognitive domains independently of HGS and traditional risk factors.

## 2. Materials and methods

### 2.1. Population

We used data from the longitudinal study in rural areas of Fuxin Mongolia Autonomous County, Liaoning province, China. This study was a part of a National key research and development program in China, which aimed to provide scientific basis for the prevention and control of chronic diseases among middle-aged and elderly people (≥35 years) in northeast China. The baseline survey was conducted in 2019, which included standardized procedures for resident questionnaire survey, health examination, and collection of blood samples from participants. A comprehensive follow-up was planned every 2 years, including health examination and collection of biological samples. Specifically, from June to August 2019, we selected 4 townships (Daban Township, Furong Township, Guohua Township, and Fuxin Township) and a total of 33 villages under them as units to recruit participants continuously in Fuxin Mongolia Autonomous County. The selection of townships and villages was jointly determined through expert discussions and consultations with the local health department, which considered population characteristics (Han and ethnic minority) and geographic distribution (eastern, southern, and northern regions). All villages under the coverage of each village doctors (the principal person in charge of health services in village) from township health center were included. The inclusion criteria for participants included: (1) ≥35 years old; (2) a local residence time ≥5 years; and (3) signed informed consent. The exclusion criteria included: (1) pregnancy; (2) severe liver and renal failure; and (3) unwillingness to participate in this study or voluntarily withdrew from the investigation. Until now, two waves of surveys have been conducted, with 4,689 participants recruited in 2019 (baseline) and 3,482 in 2021. Data was collected using standardized questionnaires and recorded by trained investigators. Informed consent was obtained from all participants. If the participants were unable to write, their guardians read and signed the informed consent form on their behalf. The study was performed under the ethical standards of the responsible committee on human experimentation of China Medical University [(2018)083].

For the current study, 6,266 participants who attended one of these two waves for the first time were included (we excluded the 2021 data from participants who were followed, and used their 2019 data). Because our study focused on asymmetric HGS and cognitive decline with aging, we selected people ≥50 years and who completed Montreal cognitive assessment (MoCA) independently. Then, participants with neurological disorder, brain injury or stroke, without HGS data (e.g., people with orthopedic disease or other severe disease, or missing data), missing covariates and with definition of ambidextrous were excluded.

### 2.2. Measurement

#### 2.2.1. Cognitive assessment

Several medical students who were trained by neurological experts used the Chinese version of Montreal Cognitive Assessment-Basic (MoCA-BC) to assess the participants’ cognitive function in a separate room. MoCA-BC is a 30-point test covering nine cognitive domains (Executive function, Fluency, Orientation, Calculation, Abstraction, Delayed Recall, visuoperception, Naming, and Attention and Concentration). When a Mongolian participant was not fluent in Mandarin, the test was assisted by a village doctor or worker who can speak Mongolian and interpreted the questions and answers. According to the previous study (Chen et al., 2016), the optimal cutoff scores for mild cognitive impairment (MCI) detection were 19 for individuals with 6 or fewer years of education, 22 for those with 7–12 years of education, and 24 for those with more than 12 years of education.

#### 2.2.2. Assessment of the handedness

Participants’ dominant hand was assessed by a 10-items handedness questionnaire prior to the HGS test: (1) Which hand do you use to write? (2)… hold the chopsticks? (3)… throw things? (4)… brush your teeth? (5)… cut things with scissors? (6)… light a match? (7)… thread the needle? (8)… hit with a hammer? (9)… play ping-pong or other sports? (10)… wash your face? The subjects were asked to answer one of three options (“use my left hand a lot,” “use my right hand a lot” or “are all often used”) that best matched their situations. If all answers to questions 1−6 were “right-handed,” the dominant hand was the right hand; if they are all “left-handed,” the dominant hand was the left hand; in the remaining cases, those who wrote or used chopsticks with their right hands but the other questions are ≥8 answers of “left-handed,” the dominant hand was judged to be the left hand, otherwise, other cases were judged to be ambidextrous. The criteria were based on the previous Chinese research, because some parents would “correct” the situation of using chopsticks or write with the left hand (Xintian, 1983).

#### 2.2.3. Assessment of HGS

Handgrip strength was measured in kilograms (measured to the nearest 0.1 kg) using a dynamometer (Jamar Plus+, Patterson Medical, United States). Rapid force was required during the measurement, and the subjects were instructed to grasp the gripper with maximum force and press it for 3 s in a standard sitting position. Starting with the non-dominant hand, each hand was measured three times in turn, 30 s apart after each measurement. Finally, the final results were recorded at the end of the test. In the present analysis, the HGS was defined as the highest recorded HGS value from either hand.

#### 2.2.4. HGS asymmetry

The HGS ratio was calculated as the maximal non-dominant HGS divided by the maximal dominant HGS. Asymmetric HGS was defined according to the “10% rule,” which meant the general 10% difference in HGS between dominant and non-dominant hand (Armstrong and Oldham, 1999). Asymmetric dominant HGS was defined as an HGS ratio below 0.9, whereas asymmetric non-dominant HGS was defined as an HGS ratio above 1.1.

#### 2.2.5. Definition of other variables

Data on demographic characteristics (age, sex, ethnicity, educational level), lifestyle factors (smoking status, alcohol consumption, and physical labor level) were collected through face-to-face interviews with standardized questionnaires. According to WHO’s definition of smoking status in 1997 (Pan American Health Organization World Health Organization, 1998), smoking status in this study was defined as categorical variables (“Non-smoking,” “Former smoking” and “Current smoking”). Smokers were defined as those who had ever smoked at least one cigarette per day and continued for at least 6 months. Smokers were classified as “Former smoking” or “Current smoking” based on whether they had quit for more than 6 months. According to “the Physicians’ Guide to Helping Patients with Alcohol Problems,” which National Institute on Alcohol Abuse and Alcoholism (NIAAA) (1995), alcohol intake in this study was defined as categorical variables (“Non-drinking,” “Former drinking” and “Current drinking”). Drinkers was defined as at least three drinks per week for 6 months. Then, drinkers were classified as “Former drinking” or “Current drinking” based on whether they had quit for more than 6 months.

Body mass index (BMI) was calculated as weight divided by height squared (kg/m^2^). According to the American Heart Association protocol (O’Brien et al., 1990), blood pressure (BP) was measured three times for at least 1 min after a minimum rest of 5 min between each measurement using a standardized automatic electronic BP measuring instrument (HEM-8102A/K). The participants were instructed to avoid alcohol consumption, cigarette smoking, coffee/tea, and exercise for at least 30 min before the BP measurement. The average of the three BP values was used for the final analysis and evaluation. Fasting plasma glucose and blood lipids were analyzed using the Roche Cobas 8000 C701 automatic biochemical analyzer in a central laboratory, and those methods were operated under the scope of accreditation (Gao et al., 2022).

Hypertension was defined as using antihypertensive drugs in the last 2 weeks, diastolic blood pressure (DBP) ≥90 mmHg or systolic blood pressure (SBP) ≥140 mmHg (Joint Committee for Guideline Revision, 2019). Diabetes mellitus was defined as fasting plasma glucose ≥7.0 mmol/L (≥126 mg/dL) or using hypoglycemic drugs or insulin. Lipid abnormalities were classified according to the Third Report of the National Cholesterol Education Program Expert Panel on Detection, Evaluation, and Treatment of High Blood Cholesterol in Adults final report (Expert Panel on Detection, Evaluation, and Treatment of High Blood Cholesterol in Adults, 2001). The definitions of depression and stroke were self-reported.

### 2.3. Statistical analysis

Continuous variables were presented as the means with standard deviations (SD), and categorical variables were expressed as percentages. Because of the asymmetric distribution, MoCA-BC was described as medians with interquartile ranges (IQR). ANOVA, Student’s *t*-test, Mann–Whitney *U*-test, and χ^2^ test were used to compare differences in continuous or categorical variables.

Linear regression models were fitted with restricted cubic splines (RCS) to account for the non-linear shape between HGS ratio and total MoCA score. Restricted cubic splines with three knots were sufficient for capturing non-linear patterns and avoiding over-fitting, and the number of knots was selected according to the AIC, BIC and the change of R^2^ of the models. The selection of covariates took into account of confounding variables and traditional risk factors. The former include handedness, wave, and demographic information, while the latter include HGS, lifestyle, and disease history so as to determine the independently association of HGS Asymmetry. Hierarchical modeling was used to add covariates in the following sequence: the original model was adjusted for HGS, handedness; then we added age, sex, education, ethnicity, smoking status, alcohol consumption, physical labor level and BMI, hypertension, diabetes, and dyslipidemia into the model (fully adjusted model); finally, we added interaction term between HGS ratio and ethnicity. Then, the HGS ratio was converted into asymmetric HGS according to the “10% rule,” fitted in a fully adjusted model. Given the statistical significance of the interaction term between the HGS ratio and ethnicity, all of the above analyses were again stratified by ethnicity.

For each cognitive domain, except an executive function with a score (total score is 1 point) of 0 was defined as cognitively impaired, participants who got a score within 25% quantile were defined as cognitively impaired in other domains (Supplementary Table 2). Logistic regression model was then used to determine the relationship between asymmetric HGS and impairment in each cognitive domain. Based on adjustment of traditional risk factors, asymmetric HGS and HGS (per 5 kg decrease) were adjusted for each other to show their independent effects.

Numerous studies found that a higher BMI in middle age (typically defined as 50 years or younger) was associated with an increased risk of dementia, while a higher BMI in later life was associated with a reduced risk of dementia, which called “obesity paradox.” Studies have also shown that obesity in adulthood was associated with a stronger HGS in middle age, but obesity and sarcopenia usually occured accompanied with aging in later life. Therefore, we excluded obese and malnourished patients (BMI < 18.5 and BMI ≥ 28) and re-analyzed the main results as the sensitivity analysis. Then we divided the study population into younger (50∼60 years) and older groups (≥60 years) to perform the subgroup analysis. All statistical calculations were performed using R software (version 4.1.1). A 2-side *P*-value of <0.05 was considered statistically significant.

## 3. Results

### 3.1. Participant characteristics

The inclusion and exclusion processes of the participants were shown in Supplementary Figure 1 finally, 2,969 participants were included in the analysis. Their descriptive characteristics were listed in Table 1. Participants consisted of 1,850 (62.3%) Han, 1,003 (33.8%) Mongolian, and 116 (3.9%) Manchu, with a mean age of 61.8 (7.47) years. Among these, 1,854(62.4%) had symmetric HGS, 641(21.6%) had asymmetric dominant HGS, 474(16.0%) had asymmetric non-dominant HGS, and the histograms for the HGS ratio in total and ethnic subgroups were displayed in Supplementary Figure 2. Compared with participants with symmetric HGS, participants with asymmetric HGS were older, had lower educational level, had lower physical labor level, had lower grip strength, had lower cognitive scores, and had higher prevalence of MCI. The characteristics of the participants stratified by ethnicity were listed in Supplementary Table 1. It can be seen that the main differences between ethnic groups are sex, BMI and education.

**TABLE 1:** Descriptive characteristics of the participants.

	Overall sample	Symmetric HGS	Asymmetric dominant	Asymmetric non-dominant	*P* ^a^
	*n* = 2969	*n* = 1854	*n* = 641	*n* = 474	
Age (years)	61.8 (7.47)	61.4 (7.43)	62.3 (7.54)	62.7 (7.43)	0.001
Sex (female)	1882 (63.4)	1158 (62.5)	411 (64.1)	313 (66.0)	0.322
Handedness (right)	2746 (92.5)	1732 (93.4)	568 (88.6)	446 (94.1)	<0.001
BMI (kg/m^2^)	24.65 (3.55)	24.64 (3.50)	24.86 (3.67)	24.42 (3.55)	0.125
Ethnicity					0.779
Han	1850 (62.3)	1153 (62.2)	392 (61.2)	305 (64.3)	
Mongolian	1003 (33.8)	632 (34.1)	221 (34.5)	150 (31.6)	
Manchu	116 (3.9)	69 (3.7)	28 (4.4)	19 (4.0)	
Education					0.019
≤Primary school	1298 (43.7)	787 (42.4)	282 (44.0)	229 (48.3)	
Middle school	1188 (40.0)	739 (39.9)	259 (40.4)	190 (40.1)	
≥High school	483 (16.3)	328 (17.7)	100 (15.6)	55 (11.6)	
Smoking status					0.649
Non-smoking	1852 (62.4)	1160 (62.6)	401 (62.6)	291 (61.4)	
Former smoking	875 (29.5)	551 (29.7)	188 (29.3)	136 (28.7)	
Current smoking	242 (8.2)	143 (7.7)	52 (8.1)	47 (9.9)	
Drinking					0.412
Non-drinking	2059 (69.3)	1289 (69.5)	441 (68.8)	329 (69.4)	
Former drinking	709 (23.9)	443 (23.9)	147 (22.9)	119 (25.1)	
Current drinking	201 (6.8)	122 (6.6)	53 (8.3)	26 (5.5)	
Physical labor level					0.014
Low	816 (27.5)	488 (26.3)	187 (29.2)	141 (29.7)	
Moderate	1908 (64.3)	1215 (65.5)	413 (64.4)	280 (59.1)	
High	245 (8.3)	151 (8.1)	41 (6.4)	53 (11.2)	
Hypertension (yes)	1393 (46.9)	855 (46.1)	318 (49.6)	220 (46.4)	0.302
Diabetes (yes)	417 (14.0)	266 (14.3)	94 (14.7)	57 (12.0)	0.378
Dyslipidemia (yes)	1195 (40.2)	736 (39.7)	270 (42.1)	189 (39.9)	0.550
HGS (kg)	28.98 (8.71)	29.34 (8.71)	28.78 (9.08)	27.86 (8.13)	0.004
MoCA-BC^b^	20.00 (15.00, 23.00)	20.00 (15.00, 23.00)	19.00 (15.00, 23.00)	18.00 (14.00, 22.00)	<0.001
MCI (yes)	1635 (55.1)	986 (53.2)	353 (55.1)	296 (62.4)	0.001

^a^*P*-values were calculated using a Student’s *t*-test for continuous variables and χ^2^ test for categorical variables as appropriate.

^b^MoCA-BC were presented as median (interquartile range) and *P*-value was calculated using Mann–Whitney *U*-test.

MoCA-BC, Chinese version of Montreal Cognitive Assessment-Basic; HGS, handgrip strength; BMI, body mass index; MCI, mild cognitive impairment.

### 3.2. Associations between HGS asymmetry and global cognitive function

**FIGURE 1:**
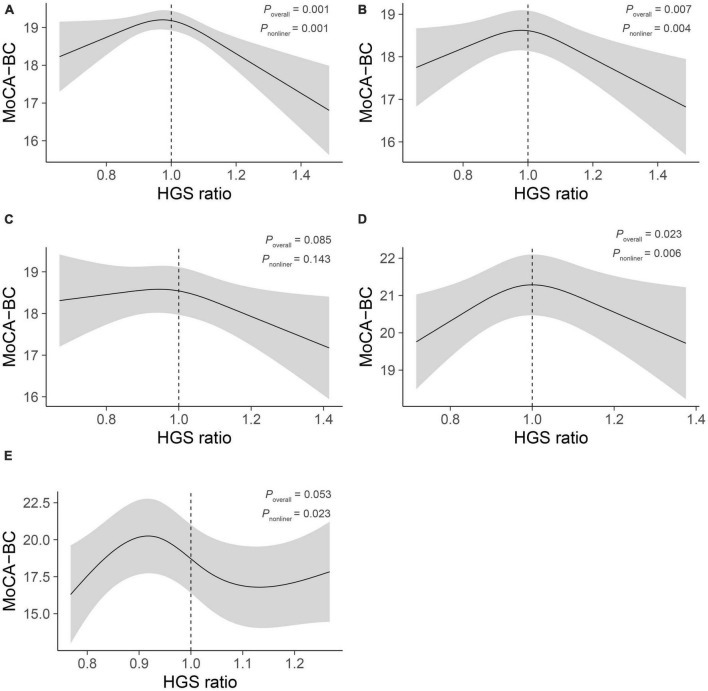
Associations between HGS ratio and global cognitive score. Linear regression models with restricted cubic splines (non-linear term) were applied to examine association between HGS ratio and the global cognitive score. Initial curve was show in panel **(A)** adjusted for HGS and handedness; then **(B)** added age, sex, wave, education, ethnicity, smoking status, alcohol consumption, physical labor level, BMI, hypertension, diabetes and dyslipidemia; **(C–E)** showed the optimum fitted curve (according to AIC, BIC, and R2) in Han, Mongolians and Manchus, separately. MoCA-BC, Chinese version of Montreal Cognitive Assessment-Basic; HGS, handgrip strength; BMI, body mass index.

We constructed all non-linear models between HGS ratio (with 3−7 knots) and global cognitive score, and the AIC, BIC, and R^2^ are shown in Supplementary Table 3. Model 1−3 finally selected 3, 3, and 4 knots, respectively. Figure 1 shows the association between HGS ratio and global cognitive score before (a: model 1) and after (b: model 2) adjustment of confounding factors. Then the interaction term of HGS ratio and ethnicity showed statistical significance (*P*_*interaction*_ = 0.048). Figures 1C–E showed the fitted curves in each ethnic groups. In Han (Figure 1C), the curve between HGS ratio and global cognitive score was flat before approximately 1, and when it was greater than 1, HGS ratio was negatively correlated with global cognitive score. In Mongolians (Figure 1D), the relationship between HGS ratio and global cognitive score followed an inverted U-shaped curve, with a HGS ratio of approximately 1 corresponding to the highest MoCA-BC score. In Manchus (Figure 1E), it also followed an inverted U-shaped curve.

The associations of asymmetric dominant/non-dominant HGS with global cognitive score in all participants were shown in Table 2 (Adjusted R^2^: 0.245). In total, asymmetric non-dominant HGS, but not asymmetric dominant HGS, was associated with lower global cognitive score (β = −0.65, 95% confidence interval [CI]: −1.13 ∼−0.17). The associations of asymmetric dominant/non-dominant HGS with global cognitive score in ethnic subgroups were shown in Table 3. In Han (Adjusted R^2^: 0.255), only asymmetric non-dominant HGS was associated with lower global cognitive score (β = −0.67, 95% CI: −1.26 ∼−0.08); in Mongolians (Adjusted R^2^: 0.228), asymmetric dominant HGS (β = −0.60, 95% CI: −1.35 ∼ 0.15) and asymmetric non-dominant HGS (β = −0.56, 95% CI: −1.42 ∼ 0.31) were all associated with lower global cognitive score, although no statistical significance was found. The association between asymmetric HGS and MCI was shown in Supplementary Table 4. In total, asymmetric non-dominant HGS, but not asymmetric dominant HGS, was associated with increased risk of MCI (OR = 1.37, 95%CI: 1.10 ∼ 1.70).

**TABLE 2:** Associations between asymmetric dominant/non-dominant HGS and global cognitive score using linear regression model.

	β (95% CI)	Standard error	*P*
(Intercept)	23.07 (20.21 ∼ 25.93)	1.46	<0.001
Age (years)	−0.16 (−0.19 ∼−0.13)	0.01	<0.001
Sex (female)	1.51 (0.88 ∼ 2.14)	0.32	<0.001
Handedness (right)	0.54 (−0.11 ∼ 1.18)	0.33	0.106
BMI (kg/m^2^)	0.06 (0.01 ∼ 0.12)	0.03	0.016
Wave (2nd)	−1.68 (−2.09 ∼−1.27)	0.21	<0.001
HGS (kg)	0.07 (0.04 ∼ 0.10)	0.02	<0.001
**HGS asymmetry**			
Symmetric HGS	1.00 (Ref.)	Ref.	
Asymmetric dominant HGS	−0.21 (−0.64 ∼ 0.22)	0.22	0.331
Asymmetric non-dominant HGS	−0.65 (−1.13 ∼−0.17)	0.24	0.008
**Ethnicity**			
Han	1.00 (Ref.)	Ref.	
Mongolian	−0.01 (−0.38 ∼ 0.36)	0.19	0.947
Manchu	1.28 (0.39 ∼ 2.17)	0.45	0.005
**Education**			
≤Primary school	1.00 (Ref.)	Ref.	
Middle school	2.58 (2.18 ∼ 2.99)	0.21	<0.001
≥High school	4.15 (3.63 ∼ 4.67)	0.27	<0.001
**Smoking status**			
Non-smoking	1.00 (Ref.)	Ref.	
Former smoking	−0.73 (−1.17 ∼−0.29)	0.23	0.001
Current smoking	−0.08 (−0.78 ∼ 0.61)	0.35	0.815
**Drinking**			
Non-drinking	1.00 (Ref.)	Ref.	
Former drinking	0.29 (−0.20 ∼ 0.79)	0.25	0.246
Current drinking	0.48 (−0.28 ∼ 1.24)	0.39	0.218
**Physical labor level**			
Low	1.00 (Ref.)	Ref.	
Moderate	−0.13 (−0.53 ∼ 0.27)	0.20	0.512
High	1.25 (0.54 ∼ 1.96)	0.36	0.001
Hypertension (yes)	−0.45 (−0.80 ∼−0.09)	0.18	0.014
Diabetes (yes)	−0.66 (−1.16 ∼−0.16)	0.25	0.009
Dyslipidemia (yes)	0.07 (−0.30 ∼ 0.43)	0.19	0.717

HGS, handgrip strength; BMI, body mass index; Ref., reference.

**TABLE 3:** Associations between asymmetric dominant/non-dominant HGS and global cognitive score in ethnic groups.

	β (95% CI)	Standard error	*P* ^a^	Adjusted R^2^
Han				0.255
Asymmetric dominant HGS^b^	0.03 (−0.51 ∼ 0.57)	0.28	0.913	
Asymmetric non-dominant HGS^b^	−0.67 (−1.26 ∼−0.08)	0.30	0.027	
HGS (kg)	0.08 (0.04 ∼ 0.12)	0.02	<0.001	
Mongolian				0.228
Asymmetric dominant HGS^b^	−0.60 (−1.35 ∼ 0.15)	0.38	0.115	
Asymmetric non-dominant HGS^b^	−0.56 (−1.42 ∼ 0.31)	0.44	0.206	
HGS (kg)	0.07 (0.01 ∼ 0.12)	0.03	0.029	
Manchu				0.185
Asymmetric dominant HGS^b^	−0.88 (−3.37 ∼ 1.60)	1.27	0.488	
Asymmetric non-dominant HGS^b^	−1.09 (−3.83 ∼ 1.66)	1.40	0.439	
HGS (kg)	−0.02 (−0.23 ∼ 0.19)	0.11	0.840	

^a^Linear regression models in each ethic group was adjusted for HGS, handedness, age, sex, waves, education, ethnicity, smoking status, alcohol consumption, physical labor level, BMI, hypertension, diabetes and dyslipidemia.

^b^Reference: symmetrical HGS. HGS, handgrip strength; BMI, body mass index.

### 3.3. Associations between HGS asymmetry and specific cognitive domains

**FIGURE 2:**
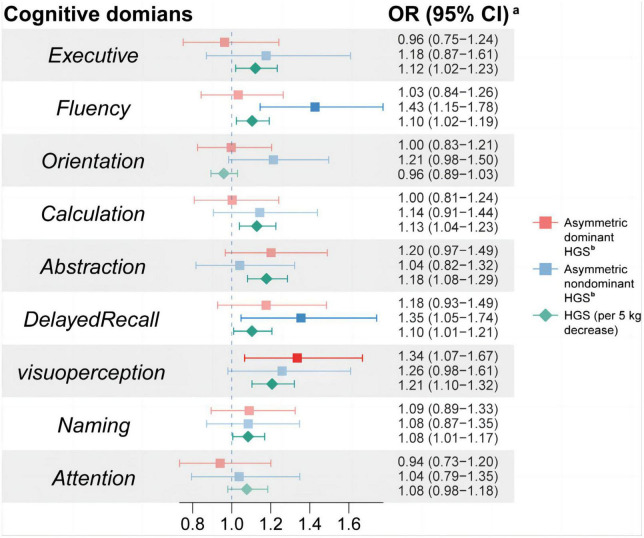
Associations between HGS asymmetry and cognitive impairment in specific cognitive domains. ^a^The logistic regression model was used to analyze the association between HGS asymmetry and impairment in various cognitive domains. Cognitive impairment in each cognitive domain was used as the dependent variable; HGS and HGS asymmetry were used as the independent variables with adjustment of age, sex, wave, education, ethnicity, dominant hand, smoking status, alcohol consumption, physical labor level, BMI, hypertension, diabetes, and dyslipidemia. HGS, handgrip strength; OR, odds ratio; CI, confidence interval.

As shown in Figure 2, asymmetric dominant HGS was independently associated with impairment of Visuoperception (OR = 1.34, 95%CI: 1.07 ∼ 1.67); asymmetric non-dominant HGS was independently associated with impairment of Fluency (OR = 1.43, 95%CI: 1.15 ∼ 1.78) and Delayed Recall (OR = 1.35, 95%CI: 1.05 ∼ 1.74). Simultaneously, per 5 kg decrease of HGS was independently associated with impairment of Executive function (OR = 1.12, 95%CI: 1.02 ∼ 1.23), Fluency (OR = 1.10, 95%CI: 1.02 ∼ 1.19), Calculation (OR = 1.13, 95%CI: 1.04 ∼ 1.23), Abstraction (OR = 1.18, 95%CI: 1.08 ∼ 1.29), Delayed Recall (OR = 1.10, 95%CI: 1.01 ∼ 1.21), Visuoperception (OR = 1.21, 95%CI: 1.10 ∼ 1.32) and Naming (OR = 1.08, 95%CI: 1.01 ∼ 1.17).

### 3.4. Sensitivity analysis and subgroup analysis

We dropped patients who were obese or malnourished and re-analyzed the association between HGS asymmetry and cognition. Supplementary Table 5 showed the association between asymmetric HGS and MoCA scores as well as the association between asymmetric HGS and MCI. The results were consistent with those found in the general population. Additionally, we found an interaction of obesity and asymmetric HGS on cognitive score (*P*_interaction_ = 0.016), with all results showing opposite associations in obese patients. Supplementary Figure 3 also showed the non-linear relationship between HGS ratio and MoCA scores among these individuals. Supplementary Figures 3E, F showed the results for the subgroups divided by age with 60 years as the boundary. It can be seen that there is still an inverted U-shaped relationship between HGS ratio and MoCA scores in the elder group (≥60 year). Supplementary Table 6 also showed that asymmetric non-dominant HGS was correlated with poorer cognitive scores (β = −1.13, 95% CI: −1.84 ∼−0.43) and a higher risk of MCI (OR = 1.73, 95%CI: 1.24 ∼ 2.44) in the elder group.

## 4. Discussion

We explored the relationship between HGS asymmetry and cognitive function in 2,969 participants based on a large-scale epidemiological survey. The principal results of our study revealed that, independent of traditional risk factors and HGS, (1) there was an inverted U-shaped association between HGS ratio and global cognitive function; (2) Overall, there was a stable association between asymmetric non-dominant HGS and poorer cognition; (3) asymmetric non-dominant HGS was independently associated with impairment of Delayed Recall and Fluency, and asymmetric dominant HGS was independently associated with impairment of Visuoperception.

McGrath et al.’s (2020) and Chen Z. et al.’s (2022) study explored asymmetric HGS (according to the “10% rule”) and weakness were associated with longitudinal cognitive changes and events of neurodegenerative disorders, respectively. The results of our study are consistent with these previous studies. Despite numerous studies to quantify the differences between the strength of the dominant and non-dominant hand (Jarjour et al., 1997; Zverev and Kamadyaapa, 2001; Yielder et al., 2009), this “10% rule” has been controversial (Petersen et al., 1989). Therefore, our study also analyzed the non-linear association between HGS ratio and cognitive score to explore more accurate U-shaped correlations. Although it is not shown in the results, we have tried the threshold effect analysis in our population, and the most sensitive threshold fluctuates somewhat (not always 1.1 and 0.9). Indeed, even we know that both low and high HGS ratios are associated with poor cognitive performance (in Mongolian group), no statistical significance still occurs when the “10% rule” is applied. Recently, a cross-sectional study of 330 community-dwelling adults found that low HGS, but not asymmetry, was independently associated with immediate and delayed memory; low HGS in combination with HGS asymmetry was associated with poorer language score (Chen K. et al., 2022). However, as mentioned in this article, they did not separate dominant and non-dominant HGS asymmetry due to the limited sample size. Based on a large-scale epidemiological survey, our study showed consistent results with them in the domains of language (Fluency). In addition, we found that asymmetric non-dominant HGS and lower HGS were all independently associated with impairments of Delayed Recall. Interestingly, previous studies have found associations between lower HGS and impairment of delayed memory (Liu et al., 2021; Jin et al., 2022), but these studies only used single side of HGS or average HGS, without HGS asymmetry. It now appears that asymmetric HGS and lower HGS should be considered together. Some circumstantial evidence suggests that the association between HGS and cognition may be due to common factors associated with aging (Fritz et al., 2017) (oxidative stress, inflammation, hormonal levels and so on). Hypothesis called “Massive redeployment” provides us with a different perspective (Anderson, 2007a,b). This hypothesis proposes that cognitive evolution proceeds in a way analogous to component reuse in software engineering, where higher-level cognition evolves by redistributing and assembling the associated low-level structures, but not by generating new structural regions. As a result, these low-level regions of the brain (related to HGS, HGS asymmetry and handedness) may be involved in various cognitive fields. These assumptions have been demonstrated in numerous studies (Heuninckx et al., 2005). The HGS asymmetry may reflect impairments in hemispheric activation, which are distinct from maximal HGS, and it is specially related to decline of memory and Language. This may because the dominant hand often participates in daily activities, especially complex and fine activities (such as writing, using chopsticks and sewing). And advanced cognitive function such as memory learned or practiced partly through those activities (Yoo et al., 2013). Therefore, decline in dominant HGS (resulting in asymmetric non-dominant HGS) may reflect cognitive decline of this areas. Our study found this independent association at the population-based level, which provided a clue for future exploration of the mechanism.

Our study found only asymmetric non-dominant HGS was associated with cognition in Han, whereas the analysis yielded clear inverted U-shaped associations in Mongolians and Manchus. McGrath’s study showed that both asymmetric non-dominant HGS (OR = 1.27, 95% CI: 1.06, 1.52) and asymmetric dominant HGS (OR = 1.12, 95% CI: 1.01, 1.25) were all associated with cognitive decline (McGrath et al., 2020). The stable association between asymmetric non-dominant HGS and poorer cognition can be explained by the more participation in complex daily activities with dominant hand mentioned-above. However, the current research’s evidence can’t explain the mechanism that the correlation between asymmetric dominant HGS and cognitive performance in the Han (no association was found) is weaker than that in the Mongolian. Chinese Mongolian people share almost the same ancestry, habits and genes with other Mongolians, which is different from Han (Jin et al., 1994; Bai et al., 2018). The differences in genetic phenotypes (e.g., the apolipoprotein E ε4 allele), dietary habits and lifestyle among ethnic groups should be investigated in the future. These may provide clues to explain the ethnic differences in association between asymmetric HGS and cognition.

Our study once again provides evidence in favor of the assessment of both hand and handedness in HGS measurement. A number of randomized controlled trials showed that multicomponent interventions improved the cognitive impairment in older adults, including exercise training (Bernabei et al., 2022; Casas-Herrero et al., 2022). Our study provides a new marker for these interventions and suggests that future interventions could be tailored to improve cognitive performance in patients with asymmetric HGS (e.g., strengthen the HGS on the asymmetric side or practice flexibility of both hands).

Our study has the following advantages: (1) Our large population-based study allowed us to fit more complex models (distinguishing asymmetric dominant/non-dominant HGS, non-linear model and interactions, etc.) so that we can take a closer look at the link between HGS asymmetry and cognition; (2) our study used a 10-question scale to determine the dominant hand, and we took into account the problem of correcting the dominant hand in the Chinese population, which allowed us to determine the dominant hand more accurately and objectively. Several limitations of our study should be noted. Firstly, our results cannot prove causality due to the cross-sectional design. However, there have been very few longitudinal studies yet, and we provide some clues for future studies. Secondly, we only used MoCA-BC to assess cognitive function. Each cognitive domain consists of only one question and may not be evaluated in detail. However, it involves 9 cognitive domains and our results provide clues for future research. In addition, MoCA-BC is a reliable, valid cognitive tool to screen for MCI in Chinese elderly population. Thirdly, during the investigation, a very small number of Mongolians did not understand Mandarin Chinese, which may cause the reliability of the MoCA scale evaluation results. But, to reduce the bias of cultural and linguistic differences among these Mongolians, investigations were carried out with the help and translation of local Mongolian speaking village doctors. These village doctors have received varying degrees of medical training, and have played a greater role in primary healthcare, including chronic disease management. They are fluent in both Mandarin Chinese and Mongolian, and have undergone strict training and assessment before being allowed to participate in the survey. Therefore, our results are relatively reliable. Finally, the small sample of Manchu is also one of limitations in this study. Therefore, results about comparing Manchu with other ethnic groups are not statistically significant. Future studies should be validated across a wider range of ethnic groups and larger samples in China.

## 5. Conclusion

In conclusion, our study found that both lower HGS and HGS asymmetry were all independently associated with lower cognitive function and MCI in a cross-sectional study of multi-ethnic population in rural China. HGS asymmetry could complement HGS, and improve the prognostic ability of HGS measurements on MCI, which suggested that future studies should consider both HGS and HGS asymmetry. Further longitudinal studies are needed to promote the application of HGS asymmetry in HGS measurement and even assessment protocols of sarcopenia.

## Data availability statement

The datasets presented in this article are not readily available because the data that support the findings of this study are restricted, which were used under license for the current study and thus are not publicly available. Requests to access the datasets should be directed to LZ, liqiangzheng@126.com.

## Ethics statement

This study was conducted in accordance with the Declaration of Helsinki and approved by the Human Experimentation of China Medical University [(2018)083]. The patients/participants provided their written informed consent to participate in this study.

## Author contributions

LZ, WF, YZ, and MM conceived and designed the study. WF, HaG, WY, RL, HuG, and CG collected the data. WF prepared the manuscript and analyzed the data. LZ and YZ had the primary responsibility for the final content. All authors reviewed the manuscript, read, and approved the final manuscript.
