# Supplementary material for: Association between handgrip strength asymmetry and cognitive function across ethnicity in rural China: a cross-sectional study
Source: Front Aging Neurosci. 2023 May 19;15:1191197. doi: 10.3389/fnagi.2023.1191197 (PMC10235480; doi:10.3389/fnagi.2023.1191197)
Supplement: Supplementary file 1 [file Table_1.DOCX]

Supplementary Material

Association between handgrip strength asymmetry and cognitive function across ethnicity in rural China: a cross-sectional study

Wenjing Feng^1,2†^, Mingfeng Ma^3†^, Hanshu Gao^1,2^, Wei Yuan^1^, Ruixue Li^1^, Hui Guo^1^, Cuiying Gu^1^, Zhaoqing Sun^4^, Yao Zhang^5*‡^, Liqiang Zheng^1,2*‡^

*** Correspondence:** Liqiang Zheng: [liqiangzheng@126.com](mailto:liqiangzheng@126.com); Yao Zhang: [cmuzhangyao@163.com](mailto:cmuzhangyao@163.com)

| **sTable 1. Descriptive characteristics in different ethnic groups** | | | | | | | |
| --- | --- | --- | --- | --- | --- | --- | --- |
|  |  | | Overall | Han | Mongolian | Manchu | ***P*** ^a^ |
|  |  | | N=2969 | n=1850 | n=1003 | n=116 |  |
| **Age (y)** | | | 61.8 (7.5) | 61.7 (7.5) | 62.0 (7.4) | 61.0 (7.6) | 0.327 |
| **Sex(famale)** | | | 1882 (63.4) | 1146 (61.9) | 652 (65.0) | 84 (72.4) | 0.032 |
| **Handedness (right)** | | | 2746 (92.5) | 1702 (92.0) | 937 (93.4) | 107 (92.2) | 0.387 |
| BMI (kg/m2) | | | 24.65 (3.55) | 24.49 (3.53) | 25.00 (3.54) | 24.21 (3.58) | <0.001 |
| **Education** | | |  |  |  |  | <0.001 |
|  | | ≤Primary school | 1298 (43.7) | 858 (46.4) | 389 (38.8) | 51 (44.0) |  |
|  | | Middle school | 1188 (40.0) | 740 (40.0) | 405 (40.4) | 43 (37.1) |  |
|  | | ≥ High School | 483 (16.3) | 252 (13.6) | 209 (20.8) | 22 (19.0) |  |
| **Smoking** | | |  |  |  |  | 0.234 |
|  | | Non-smoking | 1852 (62.4) | 1125 (60.8) | 651 (64.9) | 76 (65.5) |  |
|  | | Previous smoking | 875 (29.5) | 566 (30.6) | 276 (27.5) | 33 (28.4) |  |
|  | | Current smoking | 242 (8.2) | 159 (8.6) | 76 (7.6) | 7 (6.0) |  |
| **Drinking** | | |  |  |  |  | 0.013 |
|  | | Non-drinking | 2059 (69.3) | 1303 (70.4) | 664 (66.2) | 92 (79.3) |  |
|  | | Previous drinking | 709 (23.9) | 420 (22.7) | 268 (26.7) | 21 (18.1) |  |
|  | | Current drinking | 201 (6.8) | 127 (6.9) | 71 (7.1) | 3 (2.6) |  |
| **Physical labor level** | | |  |  |  |  | 0.911 |
|  | | Low | 816 (27.5) | 515 (27.8) | 270 (26.9) | 31 (26.7) |  |
|  | | moderate | 1908 (64.3) | 1186 (64.1) | 645 (64.3) | 77 (66.4) |  |
|  | | High | 245 (8.3) | 149 (8.1) | 88 (8.8) | 8 (6.9) |  |
| **Hypertension, yes/no** | | | 1393 (46.9) | 868 (46.9) | 472 (47.1) | 53 (45.7) | 0.962 |
| **Diabetes, yes/no** | | | 417 (14.0) | 240 (13.0) | 159 (15.9) | 18 (15.5) | 0.096 |
| **Dyslipidemia, yes/no** | | | 1195 (40.2) | 756 (40.9) | 394 (39.3) | 45 (38.8) | 0.676 |
| **HGS (kg)** | | | 28.98 (8.71) | 29.18 (8.86) | 28.73 (8.58) | 27.94 (7.23) | 0.176 |
| **HGS ratio** | | | 0.99 (0.13) | 0.99 (0.12) | 0.99 (0.13) | 0.99 (0.14) | 0.936 |
| MoCA-BC ^b^ | | | 20.00 [15.00, 23.00] | 19.00 [15.00, 23.00] | 20.00 [15.00, 23.00] | 21.00 [17.00, 25.00] | 0.002 |
| **MCI, yes/no** | | | 1635 (55.1) | 1044 (56.4) | 542 (54.0) | 49 (42.2) | 0.009 |
| ^a^ *P* values were calculated using a Student’s t-test for continuous variables and χ2 test for categorical variables as appropriate.  ^b^ MoCA-BC were presented as median (interquartile range) and *P* value was calculated using Mann-Whitney U test.  **MoCA-BC**, Chinese version of Montreal Cognitive Assessment-Basic; **HGS**, handgrip strength; **BMI**, body mass index; **MCI**, mild cognitive impairment | | | | | | | |

| **sTable2. Impairment in each cognitive domain** | | | |
| --- | --- | --- | --- |
| **Cognitive domains** | **Original scores** **^a^** | | **Cognitive impairment** |
|  | Score | n (%) | n (%) |
| Executive | **0** | **2421 (82.7)** | 2421 (82.7) |
|  | 1 | 506 (17.3) |  |
| Fluency | **0** | **1026 (34.6)** | 1026 (34.6) |
|  | 1 | 1563 (52.8) |  |
|  | 2 | 373 (12.6) |  |
| Orientation | **0** | **24 (0.8)** | 1231 (41.5) |
|  | **1** | **12 (0.4)** |  |
|  | **2** | **27 (0.9)** |  |
|  | **3** | **62 (2.1)** |  |
|  | **4** | **230 (7.8)** |  |
|  | **5** | **876 (29.5)** |  |
|  | 6 | 1736 (58.5) |  |
| Calculation | **0** | **828 (27.9)** | 828 (27.9) |
|  | 1 | 715 (24.1) |  |
|  | 2 | 529 (17.8) |  |
|  | 3 | 892 (30.1) |  |
| Abstraction | **0** | **782 (26.4)** | 782 (26.4) |
|  | 1 | 692 (23.4) |  |
|  | 2 | 843 (28.5) |  |
|  | 3 | 646 (21.8) |  |
| Delayed Recall | **0** | **594 (20.0)** | 594 (20.0) |
|  | 1 | 307 (10.4) |  |
|  | 2 | 523 (17.6) |  |
|  | 3 | 647 (21.8) |  |
|  | 4 | 492 (16.6) |  |
|  | 5 | 401 (13.5) |  |
| visuoperception | **0** | **708 (23.8)** | 708 (23.8) |
|  | 1 | 763 (25.7) |  |
|  | 2 | 1213 (40.9) |  |
|  | 3 | 285 (9.6) |  |
| Naming | **0** | **20 (0.7)** | 1013 (34.1) |
|  | **1** | **34 (1.1)** |  |
|  | **2** | **239 (8.0)** |  |
|  | **3** | **720 (24.3)** |  |
|  | 4 | 1956 (65.9) |  |
| Attention | **0** | **307 (10.4)** | **540 (18.3)** |
|  | **1** | **233 (7.9)** |  |
|  | 2 | 378 (12.8) |  |
|  | 3 | 2040 (69.0) |  |
| **^a^** In each cognitive domain, the classes of scores in bold were defined as cognitive impairment in this domain | | | |

| **sTable3. R^2^, AIC, and BIC in models with different numbers of knots (the association between HGS ratio and global cognitive score)** | | | | |
| --- | --- | --- | --- | --- |
| Models ^a^ | Knots | AIC | BIC | R^2^ |
| **Model1** | |  |  |  |
|  | 3 | **18396.64** | **18432.62** | 0.0298 |
|  | 4 | 18397.22 | 18439.19 | 0.0303 |
|  | 5 | 18399.11 | 18447.08 | 0.0303 |
|  | 6 | 18400.31 | 18454.27 | 0.0306 |
|  | 7 | 18400.28 | 18460.24 | 0.0313 |
| **Model2** | |  |  |  |
|  | 3 | **17663.67** | **17801.58** | 0.2507 |
|  | 4 | 17665.10 | 17809.00 | 0.2508 |
|  | 5 | 17666.76 | 17816.65 | 0.2509 |
|  | 6 | 17668.73 | 17824.62 | 0.2509 |
|  | 7 | 17670.46 | 17832.35 | 0.2510 |
| **Model3** | |  |  |  |
|  | 3 | 17667.52 | **17829.41** | 0.2517 |
|  | 4 | **17664.27** | 17844.15 | 0.2541 |
|  | 5 | 17670.11 | 17867.97 | 0.2541 |
|  | 6 | 17673.71 | 17889.56 | 0.2547 |
|  | 7 | 17679.24 | 17913.08 | 0.2548 |
| Minimum AIC or BIC value in each set of models were bolded  ^a^ Hierarchical modeling was used to add covariates in models with 3 to 7 knots, in the association between HGS ratio and global cognitive score.  Model1: adjusted for HGS and handedness;  Model2: Model1+ wave, age, sex, education, ethnicity, smoking status, alcohol consumption, physical labor level, BMI, hypertension, diabetes, and dyslipidemia;  Model3: Model2+ interaction term between HGS ratio and ethnicity [‘rcs(HGS_ratio, 3) × ethnicity’]  **AIC**, akaike information criterion; **BIC**, bayesian information criterion; **HGS**, handgrip strength; **BMI**, body mass index | | | | |

| **sTable 4. Associations between asymmetric dominant/nondominant HGS and MCI** | | | |
| --- | --- | --- | --- |
|  | | ***β* (95% CI)** | ***P* ^a^** |
| **Overall** (n=2969**)** | |  |  |
|  | Symmetrical HGS | Ref. |  |
|  | Asymmetric dominant HGS | 1.09 (0.90-1.33) | 0.362 |
|  | Asymmetric nondominant HGS | 1.37 (1.10-1.70) | **0.006** |
|  | HGS (kg) | 0.98 (0.97-1.00) | **0.024** |
| **Han** (n=1850**)** | |  |  |
|  | Symmetrical HGS | Ref. |  |
|  | Asymmetric dominant HGS | 0.95 (0.75-1.22) | 0.704 |
|  | Asymmetric nondominant HGS | 1.49 (1.13-1.98) | **0.005** |
|  | HGS (kg) | 0.98 (0.96-1.00) | **0.024** |
| **Mongol** (n=1003**)** | |  |  |
|  | Symmetrical HGS | Ref. |  |
|  | Asymmetric dominant HGS | 1.36 (0.98-1.90) | **0.067** |
|  | Asymmetric nondominant HGS | 1.33 (0.90-1.96) | 0.149 |
|  | HGS (kg) | 0.98 (0.96-1.01) | 0.193 |
| **Manchu** (n=116**)** | |  |  |
|  | Symmetrical HGS | Ref. |  |
|  | Asymmetric dominant HGS | 1.60 (0.50-5.10) | 0.424 |
|  | Asymmetric nondominant HGS | 0.97 (0.26-3.43) | 0.960 |
|  | HGS (kg) | 1.03 (0.94-1.14) | 0.490 |
| ^a^ Logistic regression models were used to assess the associations between asymmetric HGS and global cognitive score overall and stratified by ethnicity, adjusted for HGS, handedness, age, sex, wave, ethnicity(for overall), smoking status, alcohol consumption, physical labor level, BMI, hypertension, diabetes, and dyslipidemia.  **HGS**, handgrip strength; **BMI**, body mass index; **MCI**, mild cognitive impairment; **Ref.,** reference | | | |

| **sTable5. The association between HGS asymmetry and cognition after** **excluding obese and malnourished patients.** | | | | | | |
| --- | --- | --- | --- | --- | --- | --- |
|  |  | **Linear regression (MoCA)** | |  | **Logistic regression (MCI)** | |
|  | | ***β* (95% CI)** | ***P* ^a^** |  | **OR (95% CI)** | ***P* ^a^** |
| **Overall** | |  |  |  |  |  |
|  | Asymmetric dominant HGS ^b^ | -0.26 (-0.74 ~ 0.21) | 0.281 |  | 1.12 (0.90-1.39) | 0.305 |
|  | Asymmetric nondominant HGS ^b^ | -0.99 (-1.52 ~ -0.46) | **<0.001** |  | 1.55 (1.21-1.99) | **0.001** |
|  | HGS (kg) | 0.08 (0.04 ~ 0.11) | **<0.001** |  | 0.98 (0.97-1.00) | **0.052** |
| **Han** | |  |  |  |  |  |
|  | Asymmetric dominant HGS ^b^ | 0.11 (-0.48 ~ 0.69) | 0.725 |  | 0.94 (0.72-1.23) | 0.653 |
|  | Asymmetric nondominant HGS ^b^ | -1.14 (-1.80 ~ -0.48) | **0.001** |  | 1.79 (1.30-2.47) | **0.000** |
|  | HGS (kg) | 0.09 (0.04 ~ 0.13) | **<0.001** |  | 0.98 (0.96-1.00) | **0.075** |
| **Mongol** | |  |  |  |  |  |
|  | Asymmetric dominant HGS ^b^ | -0.89 (-1.75 ~ -0.02) | **0.046** |  | 1.59 (1.08-2.37) | **0.020** |
|  | Asymmetric nondominant HGS ^b^ | -0.71 (-1.65 ~ 0.24) | 0.144 |  | 1.43 (0.93-2.22) | 0.108 |
|  | HGS (kg) | 0.06 (-0.01 ~ 0.12) | 0.096 |  | 0.98 (0.96-1.01) | 0.307 |
| **Manchu** | |  |  |  |  |  |
|  | Asymmetric dominant HGS ^b^ | -1.71 (-4.37 ~ 0.96) | 0.213 |  | 2.26 (0.61-8.83) | 0.228 |
|  | Asymmetric nondominant HGS ^b^ | -1.14 (-4.01 ~ 1.74) | 0.441 |  | 0.85 (0.19-3.55) | 0.830 |
|  | HGS (kg) | 0.06 (-0.14 ~ 0.27) | 0.549 |  | 1.00 (0.90-1.10) | 0.968 |
| **^a^** Models adjusted for HGS, handedness, age, sex, wave, education(for linear regression), ethnicity(for overall), smoking status, alcohol consumption, physical labor level, BMI, hypertension, diabetes, and dyslipidemia.  **^b^** Reference: symmetrical HGS  **HGS**, handgrip strength; **BMI**, body mass index; **MoCA-BC**, Chinese version of Montreal Cognitive Assessment-Basic; **MCI**, mild cognitive impairment | | | | | | |

| **sTable6. The association between HGS asymmetry and cognition in younger group and older group excluding obese and malnourished patients.** | | | | | | |
| --- | --- | --- | --- | --- | --- | --- |
|  |  | **Linear regression (MoCA)** | |  | **Logistic regression (MCI)** | |
|  | | ***β* (95% CI)** | ***P* ^a^** |  | **OR (95% CI)** | ***P* ^a^** |
| **Age < 60 year** | |  |  |  |  |  |
|  | Asymmetric dominant HGS **^b^** | -0.63 (-1.32 ~ 0.07) | 0.077 |  | 1.32 (0.94-1.85) | 0.108 |
|  | Asymmetric nondominant HGS **^b^** | -0.48 (-1.27 ~ 0.31) | 0.235 |  | 1.29 (0.88-1.90) | 0.194 |
|  | HGS (kg) | 0.08 (0.03 ~ 0.13) | **0.002** |  | 0.97 (0.94-0.99) | **0.018** |
| **Age ≥ 60 year** | |  |  |  |  |  |
|  | Asymmetric dominant HGS **^b^** | 0.01 (-0.64 ~ 0.66) | 0.975 |  | 1.04 (0.78-1.38) | 0.810 |
|  | Asymmetric nondominant HGS **^b^** | -1.13 (-1.84 ~ -0.43) | **0.002** |  | 1.73 (1.24-2.44) | **0.002** |
|  | HGS (kg) | 0.09 (0.04 ~ 0.14) | **<0.001** |  | 0.98 (0.96-1.01) | **0.175** |
| **^a^** Models adjusted for HGS, handedness, age, sex, wave, education(for linear regression), ethnicity(for overall), smoking status, alcohol consumption, physical labor level, BMI, hypertension, diabetes, and dyslipidemia.  **^b^** Reference: symmetrical HGS  **HGS**, handgrip strength; **BMI**, body mass index; **MoCA-BC**, Chinese version of Montreal Cognitive Assessment-Basic; **MCI**, mild cognitive impairment | | | | | | |


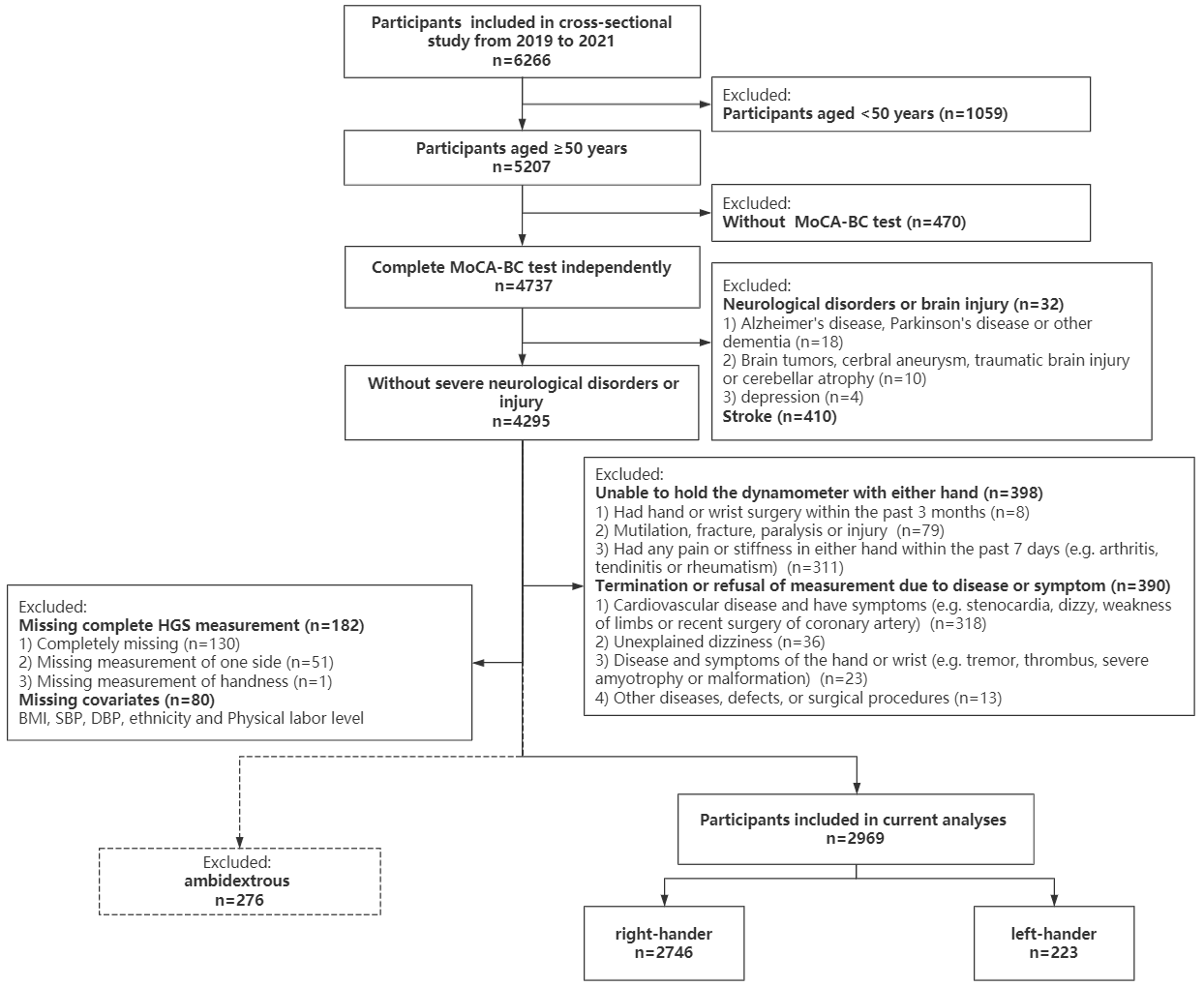
**sFigure 1. Flow diagram for the inclusion/exclusion of participants.**

**MoCA-BC**, Chinese version of Montreal Cognitive Assessment-Basic; **HGS**, handgrip strength; **BMI**, body mass index; **SBP**, systolic blood pressure; **DBP**, diastolic blood pressure


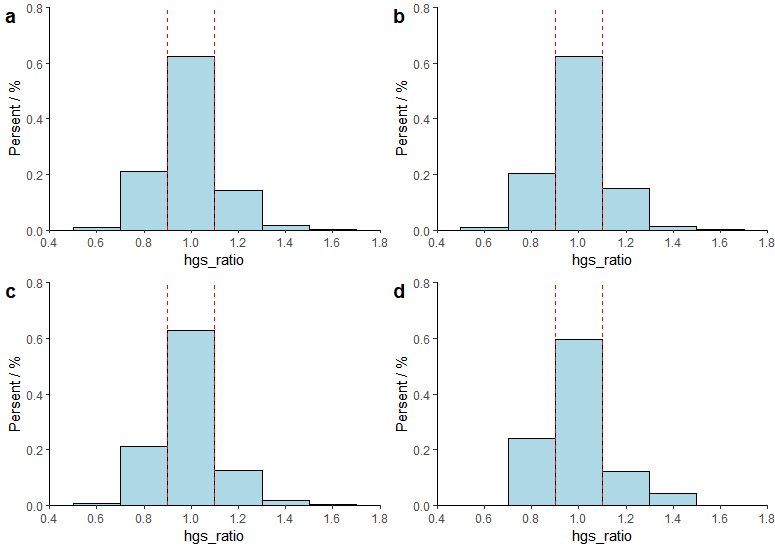
**sFigure 2. Histogram of HGS ratio ratio in overall participants (a), Han (b), Mongolian (c) and Manchu (d).**

Those with a HGS ratio below 0.9 (dominant HGS asymmetry) or above 1.1 (non-dominant HGS asymmetry) were considered to have asymmetric HGS.

**HGS**, handgrip strength


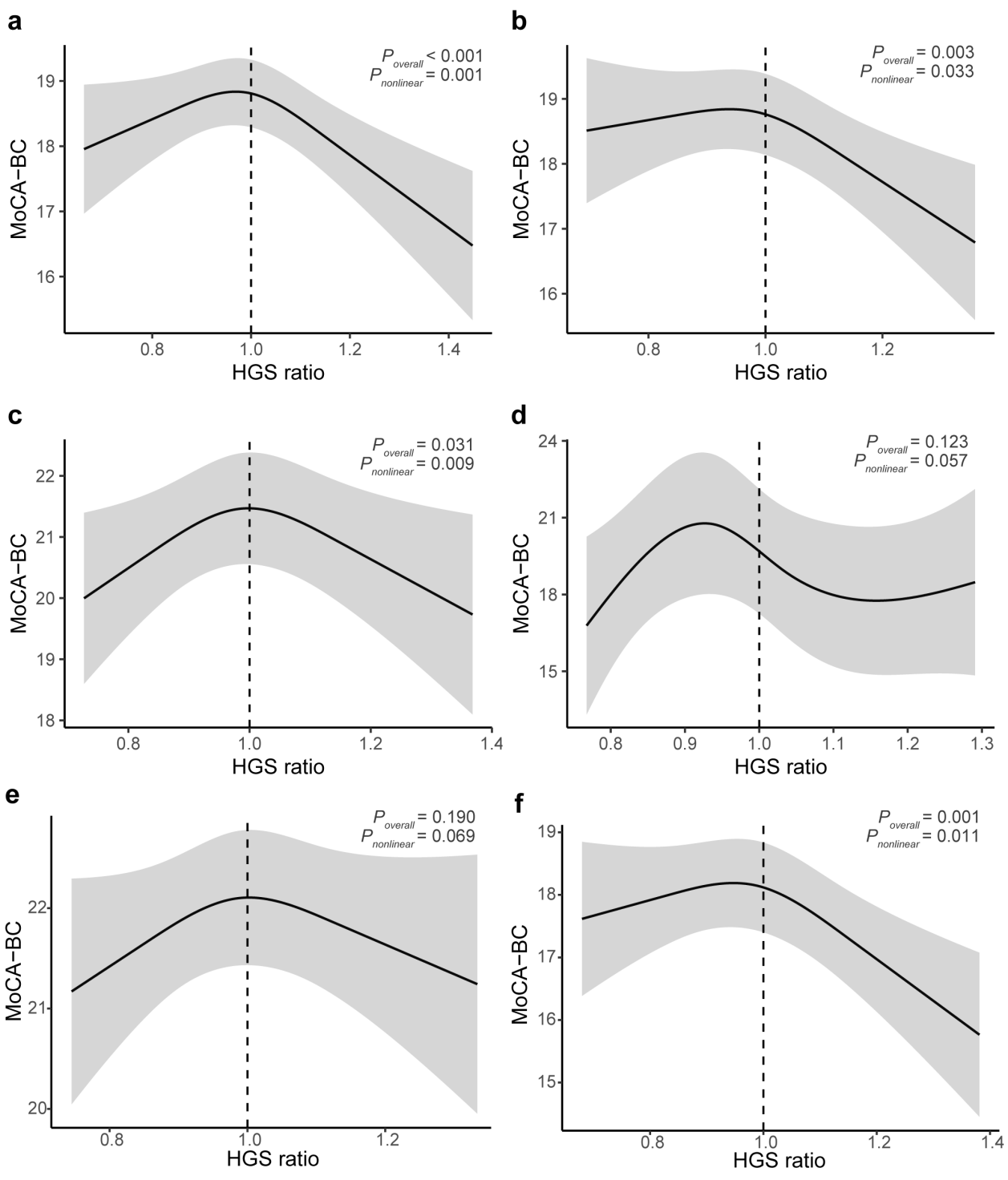
**sFigure 3. Associations between HGS ratio and global cognitive score after** **excluding obese and malnourished patients.**

After excluding obesity and malnutrition (BMI<18.5 and BMI≥28 )**,** the nonlinear association between HGS ratio and global cognitive score was shown in (a), adjusted for HGS, handedness, age, sex, wave, education, ethnicity, smoking status, alcohol consumption, physical labor level, BMI, hypertension, diabetes and dyslipidemia; (b - d) showed the optimum fitted curve (according to AIC, BIC and R2) in Han, Mongolians and Manchus, separately; (e, f) showed the association between HGS ratio and global cognitive score in younger group(e: 50 - 60 years) and older group(f: ≥ 60 years).

**MoCA-BC**, Chinese version of Montreal Cognitive Assessment-Basic; **HGS**, handgrip strength; **BMI**, body mass index
